# Trends in Dehydration in Older People: Identifying Landmark Scientific Contributions

**DOI:** 10.3390/nu17020204

**Published:** 2025-01-07

**Authors:** Olga Masot, Alexandra Pascual, Judith García-Expósito, Jéssica Miranda, Carla Camí, Teresa Botigué

**Affiliations:** 1 Department of Nursing and Physiotherapy, University of Lleida, Montserrat Roig St, 2, 25198 Lleida, Spain; olga.masot@udl.cat (O.M.); jmiranda@gss.cat (J.M.); carla.cami@udl.cat (C.C.); teresa.botigue@udl.cat (T.B.); 2 Health Education, Nursing, Sustainability and Innovation Research Group (GREISI), University of Lleida and Biomedical Research Institute of Lleida, Montserrat Roig St, 2 and Alcalde Rovira Roure Av, 80, 25198 Lleida, Spain; 3 Hospital Sant Joan de Déu Terres de Lleida, De la Canadiense Av, 28, 25001 Lleida, Spain; alexandra.pascual@sjd.es; 4 School of Nursing, University of Andorra, De la Germandat Sq, 7, AD600 Sant Julià de Lòria, Andorra; 5 Hospital Universitari Santa Maria, Gestió de Serveis Sanitaris, Alcalde Rovira Roure Av, 44, 25198 Lleida, Spain

**Keywords:** bibliometrics, dehydration, fluid intake, older people

## Abstract

Background: Dehydration and low fluid intake cause the most prevalent electrolyte imbalance in older adults and increase their morbi-mortality. Objective: To analyse the scientific production on dehydration in older adults. Methods: A bibliometric analysis was performed using the Web of Science Core Collection database. The data were analysed using two software programs, the Bibliometric package for RStudio version 4.2.2, and VOSviewer 1.6.18 for the analysis of the scientific production, authors, citations, countries and collaborations, journals, research areas, and keywords. Results: A total of 205 articles were selected. An increase in the number of publications has been observed since 2012, with 2021 being the most productive year. With respect to scientific production, although the USA had the most publications, the two most prolific authors were affiliated with an institution located in the UK, with this country having the most collaborations with other countries in the development of the subject matter. The co-occurrence analysis indicated that the words with the highest occurrence were dehydration (*n* = 103), hydration (*n* = 39), prevalence (*n* = 30), mortality (*n* = 29), and thirst (*n* = 29). Conclusions: This is the first bibliometric analysis that shows the publication trends on dehydration in older adults. Although the number of publications is limited, they have increased in number in the last few years. The research trends are centred on the prevalence of dehydration and its related factors. More studies are needed that are centred on interventions to correct the problem, to help fight against the electrolyte imbalances that occur, and to reduce the morbi-mortality associated with this condition.

## 1. Introduction

Dehydration is the most common imbalance between liquids and electrolytes in older adults, and this population group is the most susceptible to it due to the physio-pathological changes that occur during aging [1]. Likewise, the inadequate intake of fluids is an important contributing factor, which makes them more vulnerable to dehydration [2]. A recent meta-analysis [3] suggests that 24% of older adults are dehydrated (osmolality > 300 mOsm/kg). The analysis according to the different sub-groups indicated that the prevalence was 34% of the residents in long-term care, while in community-dwelling older adults, it was 19%. Nevertheless, it is striking that no clear differences were found in the prevalence according to age, sex, functional or cognitive state, or the presence of diabetes, despite the fact that most of these factors are identified as being associated with dehydration [4]. In this sense, different authors blame the low quality of the evidence on the high levels of heterogeneity between studies. Lastly, they conclude that correct hydration has a high impact on the health of older adults, as dehydration in this age group is associated with a higher mortality, a worse evolution of the disease, and higher economic costs of health services [5].

Despite the large magnitude and the dire consequences to the health of older adults, some gaps in knowledge still exist, as identified by various authors who have published on the subject. More specifically, as previously mentioned, it is difficult to establish the exact prevalence of dehydration, due to the great variability of the diagnostic methods utilized, which has also been reported by other authors [6]. Likewise, there seems to be limited evidence with regard to the improvement of this condition [7]. More high-quality studies are needed that use validated methods instead of subjective ones to promote fluid intake and assess dehydration [7]. Considering the above, it is necessary to observe, analyse, and understand the trends in the scientific literature published to date. Thus, the following research question was posed: what are the trends in scientific production on dehydration in older adults? In this sense, it was believed that the best method that could be used to answer this question was a bibliometric analysis.

Alan Pritchard [8], one of the pioneers of bibliometry, defined it as “the application of mathematical and statistical models to books and other communication media”. Bibliometrics is becoming popular and is steadily increasing in health research, as it is a useful method for analysing the development of knowledge production [9]. More specifically, it is an excellent way to obtain a trustworthy, clear, and impartial quantitative estimation of scientific publications, providing a better understanding of the literature published. Nevertheless, as opposed to systematic reviews and meta-analyses, bibliometrics does not summarize the knowledge and attributes of the subject in question [10].

Given the above, the objective of the present bibliometric study is to quantitatively analyse the scientific production on dehydration in older adults. This bibliometric analysis is useful for both new researchers and leaders in the field, as it provides an overall perspective on the publications, including research trends every year and the most influential subjects, as well as the collaboration patterns, which would also have repercussions on future studies on the subject analysed.

## 2. Materials and Methods

Given the lack of a detailed guide on the methodology used to perform a bibliometric analysis, the present study was performed following the six steps described by Selva-Pareja et al. [11], grouped into three stages based on the work by Fauzi [12]: data collection, in which a bibliometric study was performed to identify relevant studies; screening, which included the establishment of eligibility criteria and collection of data from the selected studies; and analysing the data, in which analytical techniques described by Donthu et al. [13] were applied, such as performance analysis and science mapping.

### 2.1. Stage I: Data Collection

#### 2.1.1. Literature Research

To perform this bibliometric analysis, the data were collected from the Web of Science Core Collection (WOSCC) database, as it contains records on articles published in the highest impact journals worldwide, and it provides data that can be used to analyse the interconnections between the different areas of research, such as authors and citations, among others [14]. To create the search strategy, the descriptors selected were combined in the following manner: TS = (“dehydration” OR “hypernatremia” OR “fluid intake”) AND (“aged” OR “older people” OR “elderly people” OR “older adults” OR “aging”).

#### 2.1.2. Identifying Relevant Studies

During the selection of relevant articles, inclusion criteria centred on the population were established: participants aged 65 or older, or with a mean age of 65 years or higher; and the subject matter: dehydration and low fluid intake. Likewise, the articles excluded were those that only named water intake without analysing it, and those that were not original articles, such as reviews and other documents such as books, letters to the editor, editorial piece, or notes. Limits were not placed on language or publication date.

### 2.2. Stage 2: Screening

#### 2.2.1. Eligibility Criteria

To assess the quality of the articles, a filter named “document type” (article and early access), and some Web of Science categories (Supplementary Table S1) were applied. Afterwards, during the review based on the title and abstract, the filter “document type” was again used to ensure meeting the inclusion and exclusion criteria previously mentioned.

#### 2.2.2. Study Selection and Data Collection

The articles selected were independently reviewed by researchers with experience in literature reviews. First, three researchers (AP, JM, and OM) reviewed the titles and abstracts of all the studies identified in the search. The discrepancies were moderated by a fourth reviewer (TB). All the references were analysed using Rayyan web software [15]. The information related to the articles that were finally chosen was exported and managed with an Excel spreadsheet (’savedrecs.xls’). The Preferred Reporting Items for Systematic Reviews and Meta-Analyses (PRISMA) methodology [16] was followed for the article selection process.

### 2.3. Stage 3: Analysing the Data

#### 2.3.1. Performance Analysis

A descriptive analysis, also named performance analysis by Donthu [13], as well as a bibliometric analysis of the data extracted, was performed.

For the descriptive analysis of the content, the WOSCC was utilized. First, metric data related to the publications were extracted, such as total publications and year of publication. Second, the metric variables related to the number of citations, such as total citations per article and average citation per year, were considered.

In addition, the most important journals were analysed, according to the number of articles (journal impact factor, JIF), publisher, edition, research and area category, quartile for 2022 and JIF rank, as well as their areas of research.

#### 2.3.2. Science Mapping

For the data analysis, the open-source software RStudio was used, with the “Bibliometrix 4.1.0” R package, which provides a set of tools for quantitative research in bibliometry and scientometry [17]. Data such as the following were extracted: (a) metric variables related to the authors, including the most relevant authors according to the articles published, the authors’ local impact measured by H, G and M indices, and their affiliations; (b) metric variables related to the journals, such as Most Local Cited Sources; and (c) metric variables related to the keywords, including the words’ frequency over time.

In addition, VOSviewer software (version 1.6.17, Leiden University Center for Science and Technology Studies, Leiden, The Netherlands) [18] was used to perform a co-occurrence analysis of all the keywords. KeyWords Plus and the author keywords were used as the units of analysis. KeyWords Plus corresponds to the words identified by WoS in the titles of the articles [19], while the author keywords are defined by the publication authors [20].

## 3. Results

**Figure 1:**
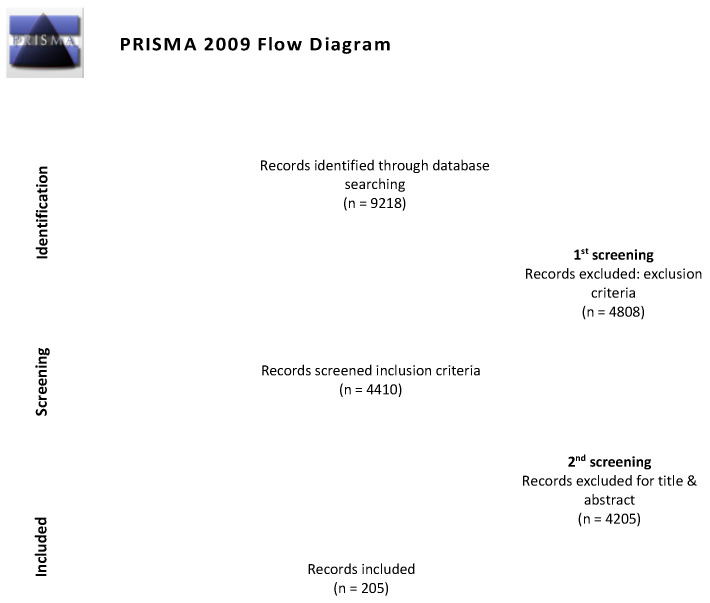
Flow diagram adapted from PRISMA [16].

The search strategy used in the WOSCC yielded a total of 9218 records. After the application of the inclusion and exclusion criteria, the resulting articles numbered 4410. Lastly, after the assessment of the titles and abstracts, a total of 205 articles were included in the analysis. The entire process is shown in the PRISMA flow diagram below (Figure 1).

### 3.1. Year of Publication and Number of Citations

**Figure 2:**
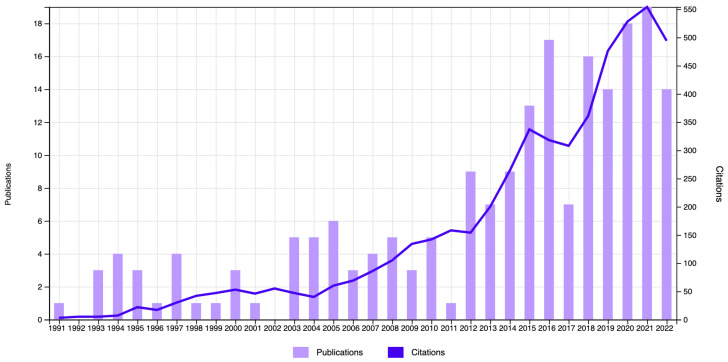
Times cited and publications over time in WOSCC (1991–2022).

The first article recorded on the subject was published in 1980, and no other records were found until 1991. Figure 2 shows the distribution of the number of publications in the WOSCC database per year and citations, from 1991 until 2022. The year 2023 was not considered, as the search took place in the middle of that year, and the information would therefore be incomplete. On the other hand, an exponential growth can be observed in the last 15 years, with 2021 being the most productive year, with a total of 19 publications.

As for the number of citations included in the bibliometric analysis, these were cited 5524 times (without including self-citations) throughout the years, with 2021 being the most cited, with a total of 554 citations. Table 1 shows the five most-cited articles. Thus, the most-cited article was published in 2003 by Hawkins [21]. However, the article by Liamis et al. [22] obtained the highest average citations per year.

### 3.2. Authors

The 205 articles included in the bibliometric study were written by a total of 1086 authors. According to the number of articles published, the most important authors were Hooper L (*n* = 10), Bunn DK (*n* = 7), Potter JF (*n* = 6), and Harvey K, Moreira P, Padrao P, Soiza RL, and Watanabe K (*n* = 4 each). Of these, the first four were also part of the groups of authors with the highest local impact according to the G, H and M indices, as shown in Supplementary Figure S1.

In addition, the first three authors were affiliated with the “University of East Anglia” (United Kingdom), which, at the same time, was found to be the organization with the highest number of publications, followed by the Pennsylvania State University and the University of California System.

### 3.3. Countries, Country Collaboration, and Publication Languages

**Figure 3:**
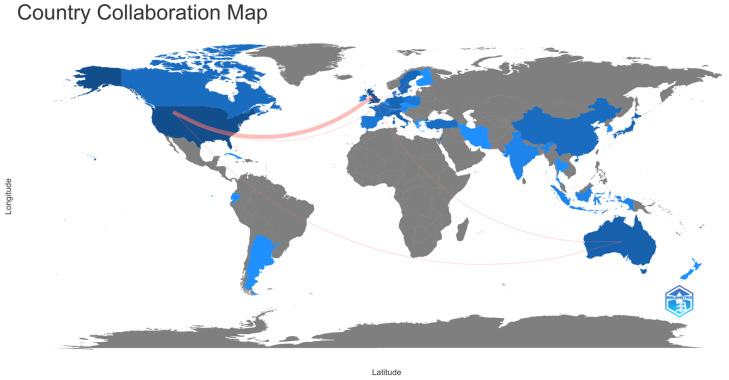
World map of countries’ collaboration.

Among the countries with the most publications on the subject, we found the USA (*n* = 49, 23.9%), the UK (*n* = 32, 15.61%), Australia (*n* = 15, 7.32%), Italy (*n* = 14, 6.83%), and Japan (*n* = 13, 6.34%). Likewise, it was observed that the countries with the highest number of collaborations were the USA and the UK, followed by the USA and the Netherlands, the USA and Australia, the UK and Sweden, the UK and Australia, and the UK and Austria (Figure 3).

This map considers all authors who have published on the topic and intensity of the colour is proportional to the number of publications. The predominant language of the publications was English, with a total of 201 articles (98%), followed by French, with two articles, and another two in Italian and Spanish, respectively. 

### 3.4. Journals and Areas of Research

A total of 129 journals published articles on dehydration in older adults, but the ones with the most articles were the following: *Nutrients* (*n* = 8), *Age and Ageing* (*n* = 7), and *Aging Clinical and Experimental Research*, *The Journal of Nutrition*, *Health and Aging*, *Journal of the American Geriatrics Society*, *Journals of Gerontology Series A: Biological Sciences and Medical Sciences*, with six publications each. According to the Journal Citation Report, the impact factor of the most prolific journals varied between 4 and 6.7, which were found to be between Q1 and Q2, and to include two areas of knowledge (Table 2).

In addition, the journals that published the most varied from the Most Local Cited sources. Among the most cited, we found *Journal of American Geriatrics Society* (*n* = 257), *Journal of American Medical Directors Association* (*n* = 145), and *The American Journal of Clinical Nutrition* (*n* = 136) (Supplementary Figure S2).

When analysing the research areas, a total of 35 different fields were observed. The fields that published the most on dehydration in older adults were the following: geriatrics and gerontology (*n* = 65, 31.71%), nutrition and dietetics (*n* = 38, 18.54%), general internal medicine (*n* = 29, 14.15%), nursing (*n* = 18, 8.78%), and neurosciences neurology (*n* = 10, 4.88%).

### 3.5. Keywords

**Figure 4:**
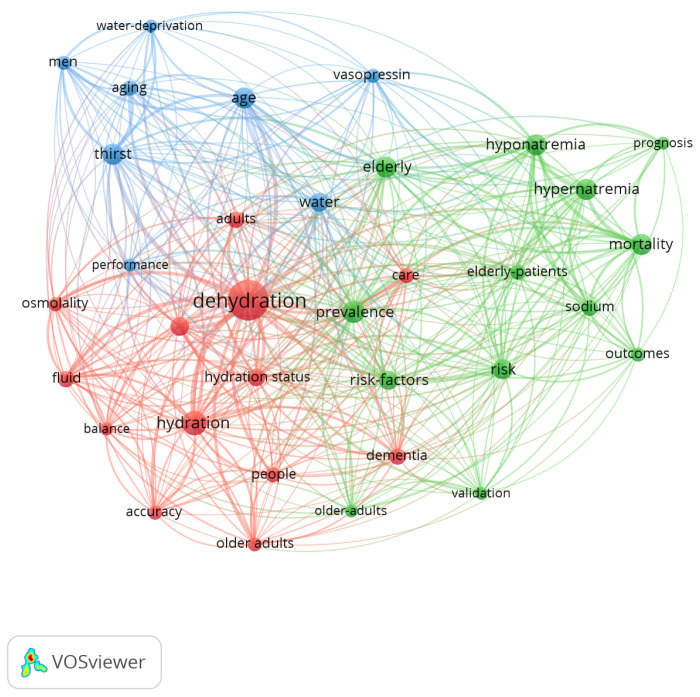
Network map of the 34 keywords with a frequency of more than 10 occurrences.

The existence of 894 keywords was determined. When examining their co-occurrence, all the keywords with a minimum of 10 occurrences were used as the units of analysis (KeyWords Plus and Author keywords), resulting in a total of 34 terms identified. In the resulting network, it was observed that the words with the highest occurrence were as follows: dehydration (*n* = 103), hydration (*n* = 39), prevalence (*n* = 30), mortality (*n* = 29), and thirst (*n* = 29) (Figure 4).

In this network map, three clusters were differentiated:

- Cluster 1 (13 items, red colour): accuracy, adults, balance, care, dehydration, dementia, fluid, fluid intake, hydration, hydration status, older adults, osmolality, and people.
- Cluster 2 (13 items, green colour): elderly, elderly patients, hypernatremia, hyponatremia, mortality, older adults, outcomes, prevalence, prognosis, risk, risk factors, sodium, and validation.
- Cluster 3 (eight items, blue colour): age, aging, men, performance, thirst, vasopressin, water, and water deprivation.

**Figure 5:**
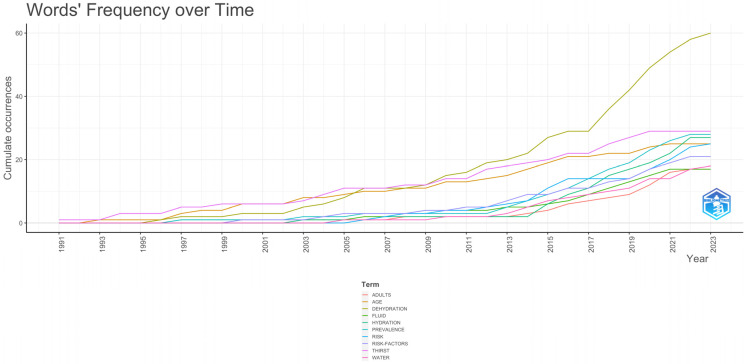
Word frequency over time.

On the other hand, when analysing the frequency of use of the keywords through time, it was observed that from 1991 to 2010, the most-utilized term was thirst, but from 2010 to the present, the most frequent word has been dehydration, followed by thirst (Figure 5).

## 4. Discussion

The results from the present bibliometric study show a global view of the scientific production on dehydration in older adults. This can be considered the first article that analyses the existing bibliographic trends on the subject, as no previous evidence was found. After the screening of the articles, 205 published manuscripts were found that met the selection criteria. This shows that this subject has been little addressed, despite its prevalence and its consequences in the older adult population group [5]. The present bibliometric analysis extracts information about the year of publication, the number of citations, authors, countries, country collaborations, languages of publication, journals, areas of research, and keywords utilized.

### 4.1. Year of Publication and Number of Citations

The first article on record on the subject was published in 1980, with the next article on record not published until 10 years later, although an exponential growth has been observed in the last 15 years. The first important peak in production was found in 2012, with nine publications, the highest number until the present. This peak could be due to the fact that two years prior, scientific organizations such as the EFSA had begun to publish scientific documents [26] granting relevance to the correct hydration of older adults. Likewise, health quality and research organizations [27] began to conduct studies on the prevention of hospitalizations among older adults. These studies showed that dehydration was a preventable condition, and it must therefore be studied. From the start of studies on the problem until the present time, the number of publications has fluctuated, although always on the rise, with 2021 being the most productive, with 19 publications. This could be due to new guidelines published in 2019 by organizations of worldwide importance such as the WHO [28] and ESPEN [29], which also coincided with the COVID-19 pandemic [30,31]. Likewise, 2021 obtained the most citations on the subject, which is related to scientific production [30,31].

As for the citations, as usual, the classical texts, such as Morley, from 1994 [23], were some of the most cited (238 citations). It must also be underlined that all the levels of healthcare where we may find older adults were represented in the five most-cited articles: hospitalization [21,24], community care [21,22,25], and nursing homes [23]. In addition, with respect to the average citation per age, the article by Liamis [22] stands out, perhaps because it is the most recent since the production boom of 2012 and includes every type of dehydration in its analysis.

### 4.2. Countries, Country Collaboration, and Publication Language

With respect to the authors who have published the most on the subject, the leading contributors are Hooper L (*n* = 10) and Bunn DK (*n* = 7). Both are part of the UK institution with the most publications. Hooper is the principal investigator of the Dehydration Recognition in our Elders project (DRIE study) [32], one of the leading projects in this area. The UK is not the country with the most publications, but it is the one with the most collaborations with other countries on this subject matter. Most of these collaborations have been with the USA, with more than 40% of the worldwide publications between them. In this sense, collaboration between countries helps researchers exchange knowledge and ideas, and it also facilitates access to resources, scientific infrastructure, and funding. The joint publications and the collaboration networks broaden the audience and the impact of the research [33]. Therefore, stronger collaboration networks must be established between countries, institutions, and authors, which will be crucial for the greater development of the subject investigated.

The main communication language between researchers from different countries is English. This, together with the fact that most of the journals that publish on this subject only accept English as the language, makes it the most-utilized language. Worldwide, English has become the dominant language in science and is found in more than 90% of the scientific articles indexed. Articles published in English have a higher number of citations than those published in other languages. This could be because the articles in English are accessible to a broader audience [34].

### 4.3. Journals and Areas of Research

Most of the publications included in the present bibliometric study were published in high-impact journals. These are high-quality journals indexed in the main citation-based index databases, such as JCR (with a JIF ranging from 4 to 6.7 points, all of which are classified in Q1 and Q2). Thus, it can be concluded that although the number of original articles on dehydration in older adults is limited, the quality of the research is high.

On the other hand, as previously mentioned, the areas of research were ‘geriatrics gerontology’, ‘nutrition and dietetics’, ‘general internal medicine’, ‘nursing’ and ‘neuroscience neurology’. Logically, due to their subject matter, geriatrics (*n* = 65, 31.71%) and nutrition (*n* = 38, 18.54%) were the two most prominent areas of publication, as they encompass the two main concepts investigated in this bibliometric study.

As for the analysis of the other areas, it is important to consider the relationship between the third area, ‘general internal medicine’ (*n* = 29, 14.15%), and the last one, ‘neuroscience neurology’ (*n* = 10, 4.88%). Different aspects have been addressed with a more acute perspective (internal medicine) related to hospitalization, where the problem is very prevalent [35,36]. More specifically, neurology is present because during aging, we find neurological situations that could compromise hydration, such as Parkinson’s disease, in 57.3% of the cases [37], and dementia, in up to 85.9% of the cases [38].

On the other hand, ‘nursing’ is the fourth area of knowledge that appeared in this bibliometric study (*n* = 18, 8.78%). Burns [39] points out that it is the nurses who mostly detect the changes in the state of the patient, based on signs and symptoms of dehydration, which are many times non-specific. Likewise, nurses focus their actions on prevention strategies, making sure to identify when the patients do not ingest a sufficient amount of fluids for health maintenance. Thus, they are also responsible for the precise recording of the ingestion of fluids by the patient, which could serve as an indicator of their state of hydration. This also helps detect the individuals with a potential risk of dehydration, prevent a delay in medical actions, and identify the causes of the unexpected deterioration of the patient. The incorrect prescription of intravenous fluids is thus avoided, as well as prolonged hospital stays and morbidity and mortality. That is, the actions of the nurses are key in the efficiency and efficacy of the care provided [40].

### 4.4. Keyword Co-Occurrence Analysis

The analysis of the different keywords revealed the existence of a great diversity of terms with a high co-occurrence. This finding highlights the multifaceted nature of dehydration as a health problem, encompassing physiological, clinical, and population dimensions. More specifically, three clusters were found regarding dehydration. The term dehydration is related to the state of hydration of people. In this cluster, we can find the term osmolality, which is considered the best gold standard available for measuring dehydration due to loss of water [6]. The importance of this parameter lies in its ability to provide an accurate quantification of hydration levels, allowing dehydration to be diagnosed and treated effectively. As for the second cluster (green), it showed a more descriptive trend of the magnitude of the problem, the associated factors, the resulting outcomes, and their prognosis (consequences). Dehydration becomes a physiological unbalancing act in which older people have more risk of suffering from acute health problems. These acute problems can be falls [24], fractures [41], acute confusion and delirium [24], pressure ulcers [42], constipation [24], and urinary infections [43]. Moreover, older people who are dehydrated are at risk of undergoing acute coronary events, pneumonia, and thromboembolism [44]. In fact, a high level of sodium is one of the main adverse prognostic indicators over a mid-term follow-up in hospitalized patients aged ≥70 years with heart failure [45]. Due to these, dehydration is associated with an increased risk of disability after four years [46]. Nevertheless, this second cluster is centred on acute dehydration, as we find terms such as ‘sodium’, ‘hypernatremia’, and ‘hyponatremia’. These types of dehydration conditions can be experienced by ill individuals of any age and are closely related to the levels of sodium in the blood. In this sense, dehydration can be classified as hypernatremia and hyponatremia, according to the relationship between the loss of liquid and electrolytes [47].

The last cluster (blue) is related to the physiopathology of dehydration, with words such as vasopressin or water deprivation. The physiological process found in a healthy adult to compensate for a reduced blood volume is a cascade of reactions known as the renin–angiotensin system [48]. Due to the pathophysiology of older people and how it affects their water homeostasis, in earlier studies, the word “thirst” was used to refer to dehydration. However, starting in 2010 (with the start of exponential growth in the number of publications), the term evolved to ‘dehydration’, as it is the final result in many conditions, among which we find the decrease in thirst. In addition, why does this alteration in thirst occur? It is a physiological aspect of the aging process and is associated with alterations in water homeostasis, which occur with advancing age. Older adults can experience diurnal fluctuations in the secretion of antidiuretic hormone (ADH) or arginine vasopressin (AVP). Thus, water homeostasis acts in parallel to restore these liquids [49]. When intracellular dehydration occurs, the osmoreceptors also signal the hypothalamus to induce the stimulation of the feeling of thirst [50]. Older adults also have a weaker sense of thirst, which is activated late, as compared to other adults. Thus, it is common to see a related hypodipsia in older adults [48]. All of this could explain the association of these keywords with the last cluster.

### 4.5. Limitations

Firstly, the bibliometric search was performed only in the WOSCC database. However, this database is considered the most representative worldwide, and it is the most utilized for bibliometric analyses [51]. Secondly, the search strategy obtained many articles related to the environment and geology. Thus, the Rayyan platform was used to ease the screening of studies by different researchers who independently applied the inclusion criteria, addressing this limitation.

## 5. Conclusions

This is the first bibliometric study that describes a detailed analysis of the publication trends in the field of dehydration in older adults. Additionally, the analysis of co-occurrence and subject evolution contribute towards the identification of the conceptual structure of the problem, providing bibliographical information relevant for predicting trends that should be the subject of future studies.

More specifically, despite the limited number of publications found on the subject, the results show an increasing trend in publications in the last few years in high-impact journals. Likewise, considering the research trends, we can affirm that an important part of the literature produced is centred on the magnitude of the problem and the accompanying factors. For this reason, it can be concluded that more research is required focusing on interventions for dehydration in older adults, to help fight against the electrolytic disequilibrium that is produced and to reduce the morbi-mortality associated with this condition. For this reason, the results provide important information to key researchers on the subject and to those that address it for the first time, stimulating new research trends and promoting collaboration among researchers across different countries, fields, and approaches.

## Figures and Tables

**Table 1:** Most-cited articles.

Article	Author	Year	Journal	Total Citations	Average Citations per Year *
Age and gender as risk factors for hyponatremia and hypernatremia [21]	Hawkins, RC	2003	Clinica Chimica Acta	276	13.14
Causes of weight-loss in community nursing-home [23]	Morley, JE and Kraenzle, D	1994	Journal of the American Geriatrics Society	238	7.93
Hypernatremia in hospitalized patients [24]	Palevsky, PM; Bhagrath, R and Greenberg, A	1996	Annals of Internal Medicine	214	7.64
Electrolyte Disorders in Community Subjects: Prevalence and Risk Factors [22]	Liamis, G; Rodenburg, EM; Hofman, A; Zietse, R; Stricker, BH; Hoorn, EJ	2013	American Journal of Medicine	199	18.09
Prevalence of Hyponatremia and Association with Mortality: Results from NHANES [25]	Mohan, S; Gu, S, Parikh, A; Radhakrishnan, J	2013	American Journal of Medicine	169	15.36

* Average number of citations per year per publication.

**Table 2:** Characteristics of the journals with the most relevance.

Journal	Publisher	JIF * 2022	Edition	Quartile2022	JIF Rank	Research Area or Category
*Nutrients*	MDPI	5.9	SCIE	Q1	17/88	Nutrition and Dietetics
*Age and Ageing*	Oxford Univ. Press	6.7	SCIE	Q1	10/54	Geriatrics and Gerontology
*Aging Clinical and Experimental Research*	Springer	4	SCIE	Q2	24/54	Geriatrics and Gerontology
*The Journal of Nutrition*, *Health and Aging*	Springer France	5.8	SCIE	Q2	14/54	Geriatrics and Gerontology
				Q1	18/88	Nutrition and Dietetics
*Journal of the American Geriatrics Society*	Wiley	6.3	SCIE	Q1	11/54	Geriatrics and Gerontology
			SSCI		4/37	Gerontology
*Journal of Gerontology Series A: Biological Science and Medical Sciences*	Oxford Univ. Press INCS	5.1	SCIE	Q2	18/54	Geriatrics and Gerontology
			SSCI	Q1	7/37	Gerontology

* Journal impact factor.

## Data Availability

The data analysed for the current study are not publicly available due to privacy restrictions but are available from the corresponding authors on reasonable request.

## Supplementary Materials

The following supporting information can be downloaded at: https://www.mdpi.com/article/10.3390/nu17020204/s1, Table S1: Web of Science Categories; Figure S1: Authors’ local impact; Figure S2: Most Local Cited Sources.
