# Supplementary material for: Trends in Dehydration in Older People: Identifying Landmark Scientific Contributions
Source: Nutrients. 2025 Jan 7;17(2):204. doi: 10.3390/nu17020204 (PMC11767598; doi:10.3390/nu17020204)

TITLE: Trends in Dehydration in Older People: Identifying Landmark Scientific Contributions

SUPPLEMENTARY DATA

Table S1: Web of Science Categories

**Web of Science Categories**
Acoustics
Agricultural economics policy
Agricultural engineering
Agriculture Dairy Animal Science
Agriculture Multidisciplinary
Agronomy
Anthropology
Archaeology
Art
Astronomy astrophysics
Automation control Systems
Biochemistry molecular biology
Biodiversity Conservation
Biology
Biotechnology applied microbiology
Cell tissue engineering
Chemistry applied
Chemistry Inorganic Nuclear
Chemistry multidisciplinary
Chemistry organic
Chemistry Physical
Computer science artificial intelligence
Computer science information Systems
Construction Building Technology
Crystallography
Demography
Development studies
Ecology
Economics
Energy fuels
Engineering Aerospace
Engineering chemical
Engineering Civil
Engineering Electrical Electronic
Engineering geological
Engineering Mechanical
Engineering Ocean
Engineering Petroleum
Ethics

Fisheries
Forestry
Genetics heredity
Geochemistry Geophysics
Geography Physical
Geology
Geosciences Multidisciplinary
Green Sustainable Science Technology
History
History Philosophy of science
Horticulture
Hospitality Leisure Sport Tourism
Humanities multidisciplinary
Immunology
Law
Limnology
Marine Freshwater Biology
Materials Science Biomaterials
Materials Science Ceramics
Materials science characterization testing
Materials science coating films
Materials Science Composites
Materials science multidisciplinary
Materials science paper wood
Materials Science textiles
Mathematical computational biology
Mathematics Applied
Mathematics interdisciplinary Applications
Mechanics
Medical Ethics
Metallurgy Metallurgical Engineering
Meteorology Atmospheric Sciences
Microbiology
Microscopy
Mineralogy
Mining Mineral Processing
Mycology
Nuclear science technology
Obstetrics Gynecology
Oceanography
Operations Research management science
Ornithology
Paleontology
Pediatrics
Physics Atomic Molecular Chemical

Physics Condensed Matter
Physics multidisciplinary
Plant sciences
Psychology applied
Public environmental occupational Health
Quantum Science Technology
Regional urban Planning
Robotics
Social science biomedical
Social sciences interdisciplinary
Soil Science
Statistics probability
Toxicology
Transportation Science Technology
Tropical medicine
Urban studies
Veterinary Sciences
Water resources
Womens Studies
Zoology

Figure S1: Authors' local impact

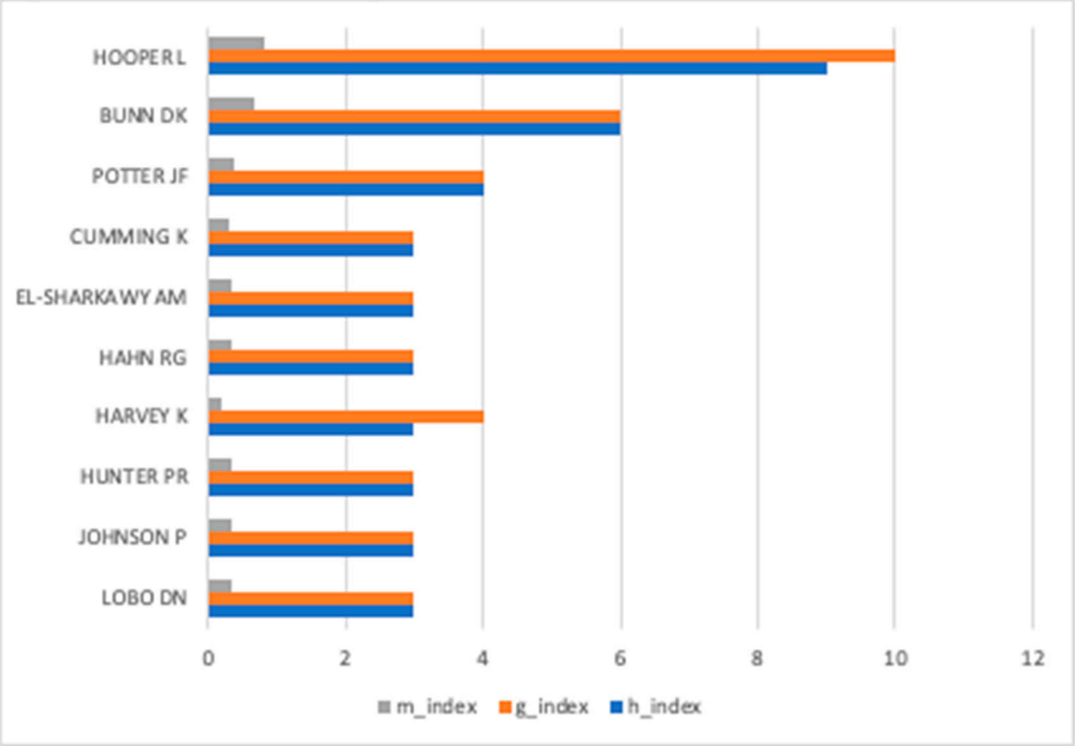

Figure S2: Most Local Cited Sources

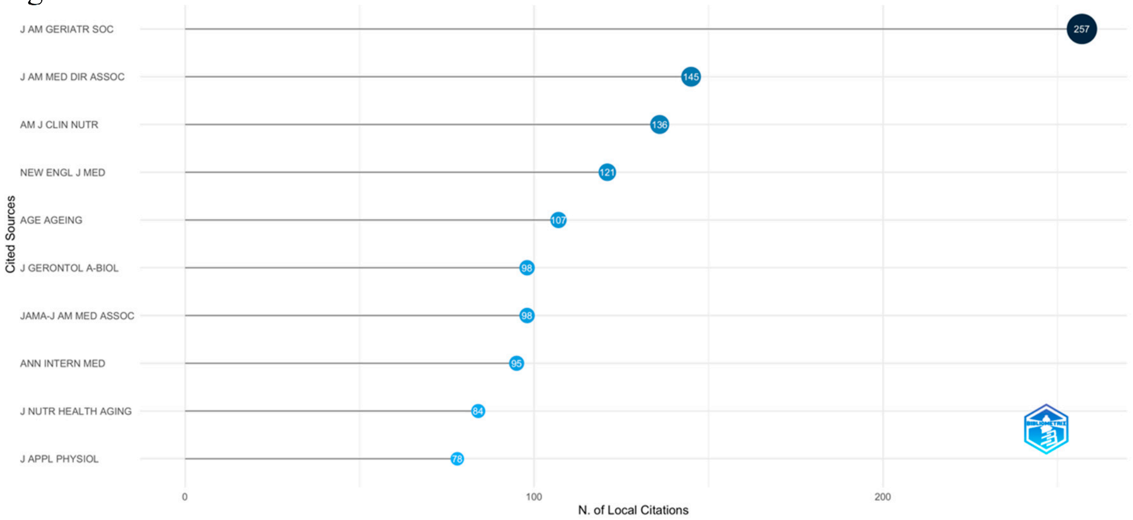

Supplement: Supplementary file 1 [file nutrients-17-00204-s001.zip › nutrients-3392644-supplementary.pdf]
